# When structure meets function

**DOI:** 10.7554/eLife.51746

**Published:** 2019-10-15

**Authors:** Sarah A Signor

**Affiliations:** Department of Biological SciencesNorth Dakota State UniversityFargoUnited States

**Keywords:** copulation, mating behavior, yellow, sex comb, melanization, *D. melanogaster*

## Abstract

A new study upturns the long-held belief that the *yellow* gene determines sex-specific behaviors in fruit flies by acting in the brain.

**Related research article** Massey JH, Chung D, Siwanowicz I, Stern DL, Wittkopp PJ. 2019. The *yellow* gene influences *Drosophila* male mating success through sex comb melanization. *eLife* **8**:e49388. doi: 10.7554/eLife.49388

It is a truism to say that in many organisms, body structure matters for behavior: jumping is not possible without legs, or flying without wings. However, scientists sometimes overlook morphology when trying to understand behavior, preferring instead to favor explanations that involve the brain and the nervous system. For instance, for decades it was thought that the *yellow* gene in fruit flies, which gives them their black color, was important for courtship behaviors because it is also expressed in the central nervous system (Drapeau et al., 2003; Drapeau et al., 2006). Male flies deficient in this gene mate less, and it was assumed that this was a consequence of changes in the neuronal wiring of the behavior controlled by *yellow*.

This makes intuitive sense in many ways because pigmentation genes such as *yellow* are derived from – and can bind to – dopamine, a chemical that has many neurological roles. However, in addition to creating color, pigments can also shape the structural properties of the external skeleton (Wittkopp and Beldade, 2009). Now in eLife, Patricia Wittkopp (University of Michigan), David Stern (Janelia Research Campus) and colleagues, including Jonathan Massey as first author, report the results of elegant experiments that rule out a neurological role for *yellow* in altering courtship behavior (Massey et al., 2019).

During courtship, male fruit flies perform a number of actions such as singing and extending their wings. Massey et al. found that the insects that lacked *yellow* also displayed the same mating behaviors, but they spent less time initiating copulation with females. This suggests that *yellow* might be important for this process, so the researchers set out to identify the types of cells in which the absence of *yellow* would have an impact on the beginning of copulation. They used two genes which regulate sex-specific behaviors and sexual dimorphism to manipulate where *yellow* was expressed in the body. *Fruitless* controls the expression of *yellow* in the central nervous system of larvae, while *doublesex* acts indirectly on *yellow* and is responsible, among other roles, for sex-specific pigmentation (Drapeau et al., 2006; Kopp et al., 2000; Williams et al., 2008; Signor et al., 2016).

First, flies were genetically engineered so that *yellow* was only expressed in the central nervous system, under the control of *fruitless*. This did not restore normal mating behavior. Massey et al. then used *doublesex* to control the expression of *yellow*. When the gene was not expressed in the tissues where *doublesex* is present, the flies failed to start mating; however, they also showed lack of mating when *yellow* was expressed in the nervous system under the control of *doublesex.* The insects only mated normally when *yellow* was expressed in other, non-neuronal cells.

To find out which non-neuronal cells might be responsible for the difference in mating success, Massey et al. examined the sequences that regulate the expression of *doublesex*, looking for regions that had an effect on reproductive behavior in male flies. A region was identified, which drove the expression of *doublesex* in the sex combs. This structure is formed of bristles on the forelegs of male flies and contains large amounts of melanin pigment. Removing the combs does not influence courtship behavior, but it does reduce mating success (Ng and Kopp, 2008). Moreover, it had been shown previously that the expression of *doublesex* is involved in the development and diversification of sex combs in fruit flies (Tanaka et al., 2011). In the latest work, Massey et al. showed that these structures are present when *yellow* is not expressed, but that they are not melanized: this prevents male flies from efficiently grasping female flies and starting to mate.

For many years, *yellow* was thought to influence courtship behavior through its expression in the central nervous system, and its role in the structural properties of the sex comb was entirely overlooked. By showing that neuronal sources of *yellow* do not affect courtship, the work of Massey et al. is both an exciting reminder that structure determines function, and a cautionary tale about the dangers of overlooking the physical aspects of behavior.
